# Targeting MDM2 affects spastin protein levels and functions: implications for HSP treatment

**DOI:** 10.1038/s41420-025-02333-y

**Published:** 2025-02-07

**Authors:** Francesca Sardina, Federica Polverino, Sonia Valentini, Claudia Carsetti, Elisabetta Falvo, Giada Tisci, Silvia Soddu, Fabiola Moretti, Alessandro Paiardini, Cinzia Rinaldo

**Affiliations:** 1 Institute of Molecular Biology and Pathology, Italian National Research Council, c/o Sapienza University, Rome, Italy; 2 Institute of Biochemistry and Cell Biology, Italian National Research Council, Rome, Italy; 3 Department of Biology and Biotechnology “Charles Darwin”, Sapienza University, Rome, Italy; 4 Department of Biochemical Sciences “A. Rossi Fanelli”, Sapienza University, Rome, Italy; 5 Unit of Cellular Networks and Molecular Therapeutic Targets, Department of Research and Advanced Technologies, IRCCS Regina Elena National Cancer Institute, Rome, Italy

**Keywords:** Neurodegeneration, Mechanisms of disease, Motor neuron disease, Ubiquitylation

## Abstract

Spastin is a microtubule (MT) severing enzyme that regulates several cell functions associated with MT dynamics. A reduction in spastin protein levels is responsible for approximately 40% of cases of Hereditary Spastic Paraplegia (HSP), a neurodegenerative disease. Currently, there is no cure for HSP but strategies to induce a recovery of spastin levels are emerging as potential therapeutic approaches. Here, we show that MDM2 interacts with spastin MT-interacting and trafficking (MIT) domain. By biochemical and functional experiments, we demonstrate that MDM2 binds spastin and regulates its levels in a post-transcriptional manner independently of the E3 ubiquitin ligase activity. Of relevance, treatment of spastin-deficient cells with the MDM2 inhibitor Nutlin-3a can restore spastin levels and functions, such as cytokinetic abscission and sorting of transferrin receptor. These findings identify MDM2 as a novel interactor of spastin and a potential druggable regulator of its protein levels.

## Introduction

HSP is a neurodegenerative motor neuron disorder that is commonly caused by haploinsufficiency of the SPG4 gene, which encodes spastin. The majority of pathogenic SPG4-HSP variants are truncating mutations leading to reduced spastin levels associated to defects in several processes affecting intracellular transport, including microtubule (MT) dynamics and receptor sorting. Several studies have demonstrated the importance of maintaining optimal spastin levels within cells [1, 2]. Therefore, strategies to induce a recovery of spastin levels are emerging as therapeutic approaches [2–5], underscoring the importance of identifying factors and pathways that impact spastin levels and functions. We recently showed that HIPK2-mediated phosphorylation of spastin promotes its stability by preventing poly-ubiquitination/degradation via the cullin4a-Ring-Ligase (CRL4a) complex [6]. In addition, we found that it is possible to induce spastin recovery and a rescue of HSP-associated defects by blocking spastin degradation through inhibition of its poly-ubiquitination or HIPK2 overexpression [7, 6].

Two major isoforms of spastin, M1 and M87, have been identified due to different translation start sites [8]. M87 is the most abundant ubiquitous form, while M1 can only be detected at low levels in neuronal tissues [3]. M1 shares the same domains as M87 but contains a specific intramembrane hydrophobic domain (HD), which allows it to predominantly localize in the endoplasmic reticulum [9]. M1 and M87 bind to MTs and form hexamers, which are essential for MT severing. Additionally, alternative splicing forms, M1Δ4 and M87Δ4 that lack the exon 4, were observed. All isoforms share common domains, including an ATPase domain preceded by a MT-binding domain (MTBD) and a MT-interacting and trafficking domain (MIT), crucial for spastin interaction with endosomal sorting complexes required for transport (ESCRT)-III proteins [10]. This last interaction is functionally relevant both during cell division and in interphase to coordinate several processes, including the cytokinetic abscission and the endosomal trafficking [11–13]. In particular, spastin-deficient cells show a delay in abscission, resulting in the accumulation of cells connected by long intracellular bridges (ICB; [12–14]) during cytokinesis, whereas, in interphase, show recycling tubules that fail to separate from the sorting endosomes, resulting in the degradation of various cargos, such as the ferritin/transferrin receptor TfR1, rather than in their return to the plasma membrane [15, 16]. TfR1 plays a critical role in iron uptake, through endocytosis of transferrin-bound iron or ferritin H-Type (FtH), a process critical for neuronal functions [17–19]. Recently, deletion of TfR1 in interneurons has been shown to induce an HSP-like phenotype in mice [20], supporting the crucial role of this receptor in HSP.

Murine double minute 2 (MDM2) is an E3 Uquitin ligase most extensively studied as a negative regulator of the p53 tumor suppressor protein [21]. However, MDM2 exhibits p53-independent activities and modulates several other targets through different mechanisms, such as transcription, post-translational modifications, protein degradation, binding to cofactors, and subcellular localization [22]. MDM2 is expressed in many organs and cells, including neurons [23, 24]. It is involved in a number of different physiological processes, including regulation of the cell cycle, stress response and neuronal plasticity. Pathologically, beside its well-documented oncogenic potential, MDM2 plays a role in numerous chronic diseases and conditions, including inflammation and autoimmune disorders, dementia, and neurodegenerative diseases [25, 26].

During a siRNA-mediated screening aimed to recovery spastin levels, we observed that MDM2 downregulation associates with increased spastin expression. Here, we show that MDM2 directly interacts with spastin and regulates its poly-ubiquitination and protein levels independently of p53, HIPK2 and MDM2 catalytic domain. Finally, we showed that pharmacological inhibition of MDM2 by Nutlin-3a increases spastin levels and reduces the defects in cytokinesis and TfR1 receptor sorting observed in spastin-deficient cells.

## Results

### MDM2 downregulation increases spastin levels

**Fig. 1:**
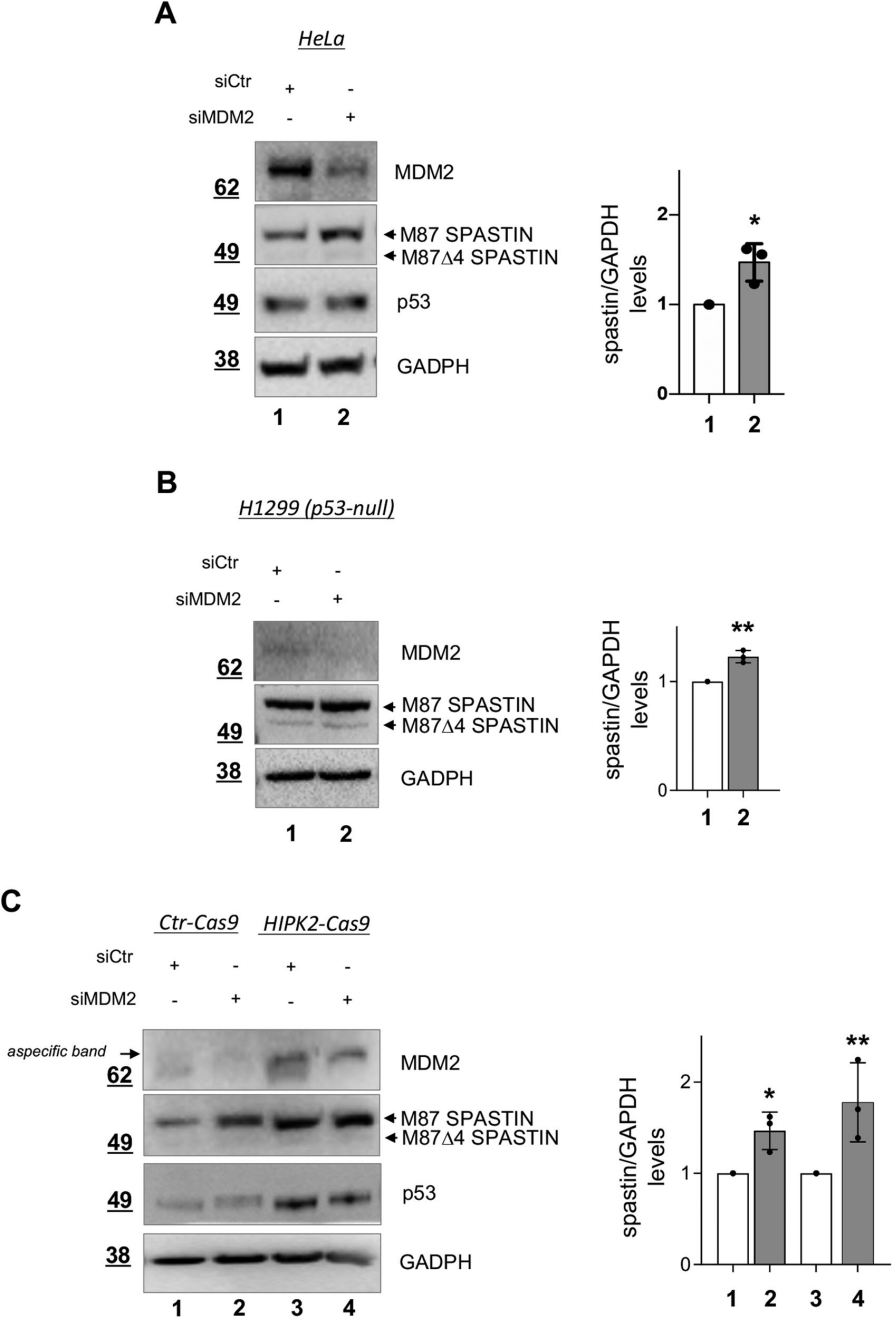
RNAi-mediated MDM2 downregulation increases spastin protein levels. **A** HeLa cells were transfected with a mixture of 20 nM MDM2-specific or negative control stealth siRNAs; cells were collected 24 h later. Representative WB with indicated Abs. The intensity of spastin bands was quantified, normalized by using the intensity of GAPDH band. And reported as mean ± SD, *n* = 3. *P < 0.05, unpaired t-test. **B** H1299 cells were transfected and analyzed as in (**A**). Spastin quantification is reported as mean ± SD, *n* = 3. **P < 0.01, unpaired t-test. **C** HIPK2-Cas9 HeLa cells and their parental control cells (Ctr-Cas9) were transfected and analysed as in (**A**). Representative WB is shown. Spastin quantification is reported as mean ± SD, *n* = 3. Statistical differences are relative to the corresponding control, *P < 0.05 and **P < 0.01, Anova test.

Based on the results from a single-siRNA-mediated screen of ubiquitination ligases involved in spastin regulation, we used a mixture of three MDM2-specific or negative control validated stealth siRNAs to confirm the MDM2 downregulation effects on spastin levels. HeLa cells were transfected and analyzed by Western blot (WB) after 24 h. An increase in spastin protein levels was observed in MDM2-downregulated (siMDM2) cells compared to control cells (siCtr) (Fig. 1A).

Beside its best-known target p53, MDM2 regulates many additional targets, including HIPK2 [27] which in turn controls spastin stability [14, 7]. Thus, we performed MDM2 downregulation by siRNA in p53-null cells, specifically in lung adenocarcinoma H1299 cells, and in HIPK2-null (HIPK2-Cas9) HeLa cells, obtained by CRISPR/Cas9 technology [28] (Fig. S1). As shown in Fig. 1B, C, a significant increase in spastin levels was observed in both p53-null and HIPK2-null cells after MDM2 downregulation, indicating that MDM2-mediated spastin regulation is independent of both p53 and HIPK2. These findings support the existence of a MDM2-mediated spastin regulatory mechanism that does not rely on the activation of the p53 network and is independent of HIPK2-mediated phosphorylation, identifying a novel pathway affecting spastin levels.

### MDM2 binds to spastin and regulates spastin poly-ubiquitination in a catalytic-independent manner

**Fig. 2:**
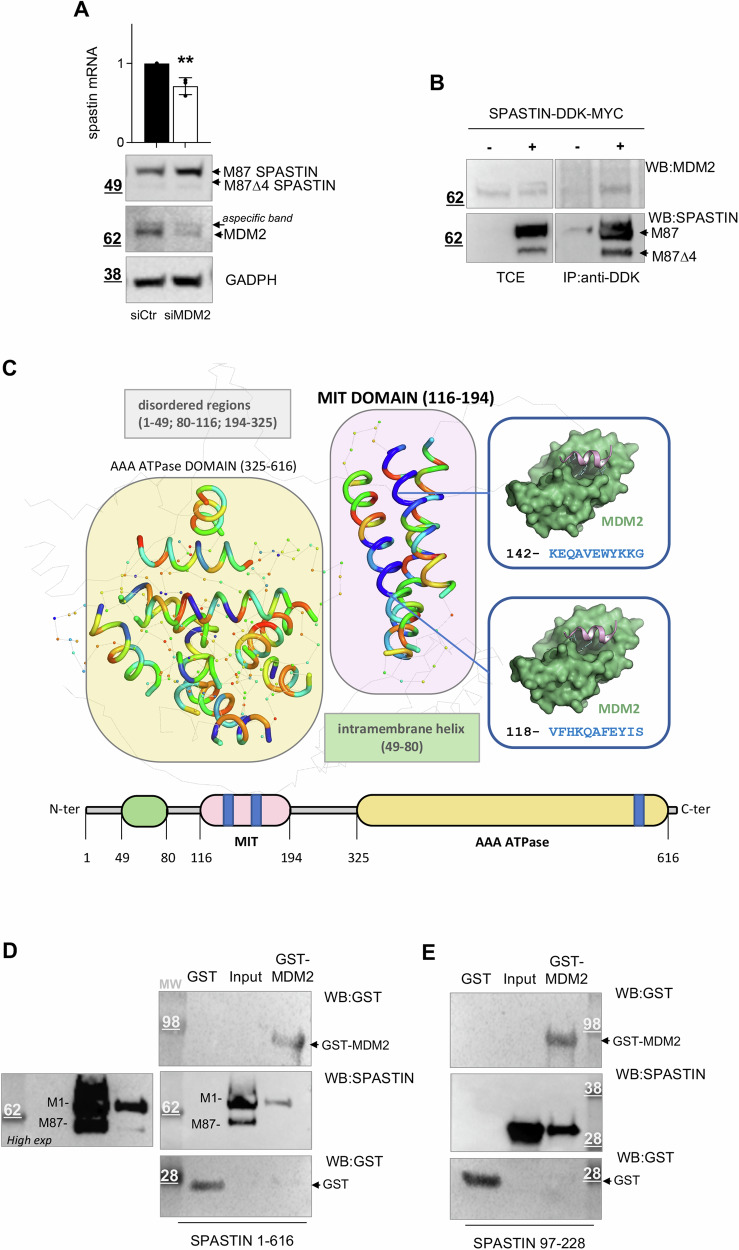
Spastin is regulated by MDM2 in a transcriptional manner and directly binds to MDM2. **A** HeLa cells were transfected as in 1B, samples were halved and analyzed by real-time RT-PCR and WB, respectively, 24 h post transfection. Relative fold-change of spastin mRNA levels, using GAPDH mRNA as normalizer, as mean ± SD *n* = 3, and representative WB are shown. **B** HeLa cells were transfected with pCMV6-DDK-MYC or pCMV6-spastin-DDK-MYC, which expresses higher levels of the M1 isoform compared to M87, consistent with data showing M1 as the most prevalent form when both isoforms have equally good Kozak’s sequences [14]. Cells were lysed 24 h post transfection and TCEs were immunoprecipitated with anti-DDK Ab and analyzed by WB with indicated Abs. Representative WB with different exposure times for TCEs and IP is shown. TCE and IP samples were loaded on the same gel and processed on the same filter. Blots were vertically cropped to show appropriate expositions. **C** Mapping of potential MDM2 helical interaction sites on the structure of spastin. The Alpha-Fold predicted structure of spastin is shown as semi-transparent grey cartoon. Structured regions are indicated with a spot, while helical regions are depicted as ribbons. The normalized rainbow scale (0–1) used to color the structured regions of spastin indicates regions of low binding affinity for MDM2 (red, 0) to high binding affinity (blue, 1). The intramembrane structured region (49–80) of spastin was excluded from the analysis. The MIT domain (region 116–194, violet) is predicted to interact with MDM2 with several high-scoring peptide regions (indicated in blue). The predicted interaction is highlighted on the right panels. In the AAA ATPase domain (region 326–616, pale orange) only a high scoring region was identified. **D**, **E** GST and GST-MDM2 proteins were bacterially expressed, purified by GST pull-down, and incubated with an equal amount of indicated recombinant spastin. Spastin binding was detected by WB. Representative WB is shown; input = 50 ng of recombinant spastin proteins (M1/M87) or recombinant spastin fragment containing the MIT domain.

MDM2 acts through a range of alternative mechanisms, including transcriptional regulation or direct interaction with its targets and subsequent negative regulation of their expression mainly, though not exclusively, through its catalytic E3 Ubiquitin ligase activity [29, 22, 30]. We first evaluated whether MDM2 transcriptionally regulates spastin by analyzing spastin mRNA levels in MDM2-downregulated and control cells. We found that the siMDM2-mediated increase in spastin protein expression does not associate with a concomitant increase of spastin mRNA levels, which rather slightly decreases upon MDM2 downregulation (Fig. 2A), excluding a MDM2-mediated transcriptional repression of spastin.

Next, we analyzed whether MDM2 interacts with spastin at the protein level by coimmunoprecipitation experiments. TCEs from HeLa cells transfected with pCMV6-DDK-MYC vector or pCMV6-spastin-DDK-MYC, expressing M1 and M87 isoforms, were immunoprecipitated with anti-DDK Ab. We found that endogenous MDM2 coimmunoprecipitates with spastin-DDK-MYC but not with DDK-MYC negative control (Fig. 2B), suggesting that MDM2 participates in cellulo complexes with spastin. Consistently, we observed co-localization of spastin-DDK-MYC and MDM2 in both the cytoplasm and the nucleus by performing in situ proximity ligation assay (isPLA) (Fig. S2A)

MDM2 is known to exhibit a high affinity for hydrophobic, helical short peptides [31, 32]. Thus, to characterize the MDM2 interaction with spastin we employed the cutting-edge AlphaFold2 [33] to obtain a reliable full-length model of spastin M1. This model highlighted regions characterized by an α-helical content and revealed that the regions with a high α-helical content were mainly concentrated in the intramembrane HD domain (49–80), the MIT domain (116–194), and the AAA ATPase domain (326–616) of spastin (Fig. 2C). Given the low probability of the helix in the intramembrane HD being implicated in the MDM2 interaction, we focused on the MIT and AAA ATPase domains. We segmented these domains into short helical peptides to identify potential helical regions that could interact with MDM2. Subsequently, we assessed their affinity for MDM2 through peptide-protein docking [34] (Fig. 2C). The analysis of the MIT domain unveiled the presence of multiple high-scoring peptide regions, predicted to interact with the p53-binding cleft of MDM2. In contrast, the AAA ATPase domain exhibited only a single high-scoring region. Consequently, the MIT domain was selected together with the full-length spastin for wet analyses. By pull-down experiments performed with purified proteins, we observed that spastin full-length isoforms (M1 and M87), spastin spliced isoforms Δ4, and a spastin fragment encompassing the MIT domain directly bind to GST-MDM2, but not to GST alone (Figs. 2D and S2B, 2E). These results show that MDM2 can directly bind spastin through the MIT domain, although it is possible that other domains may also be involved.

**Fig. 3:**
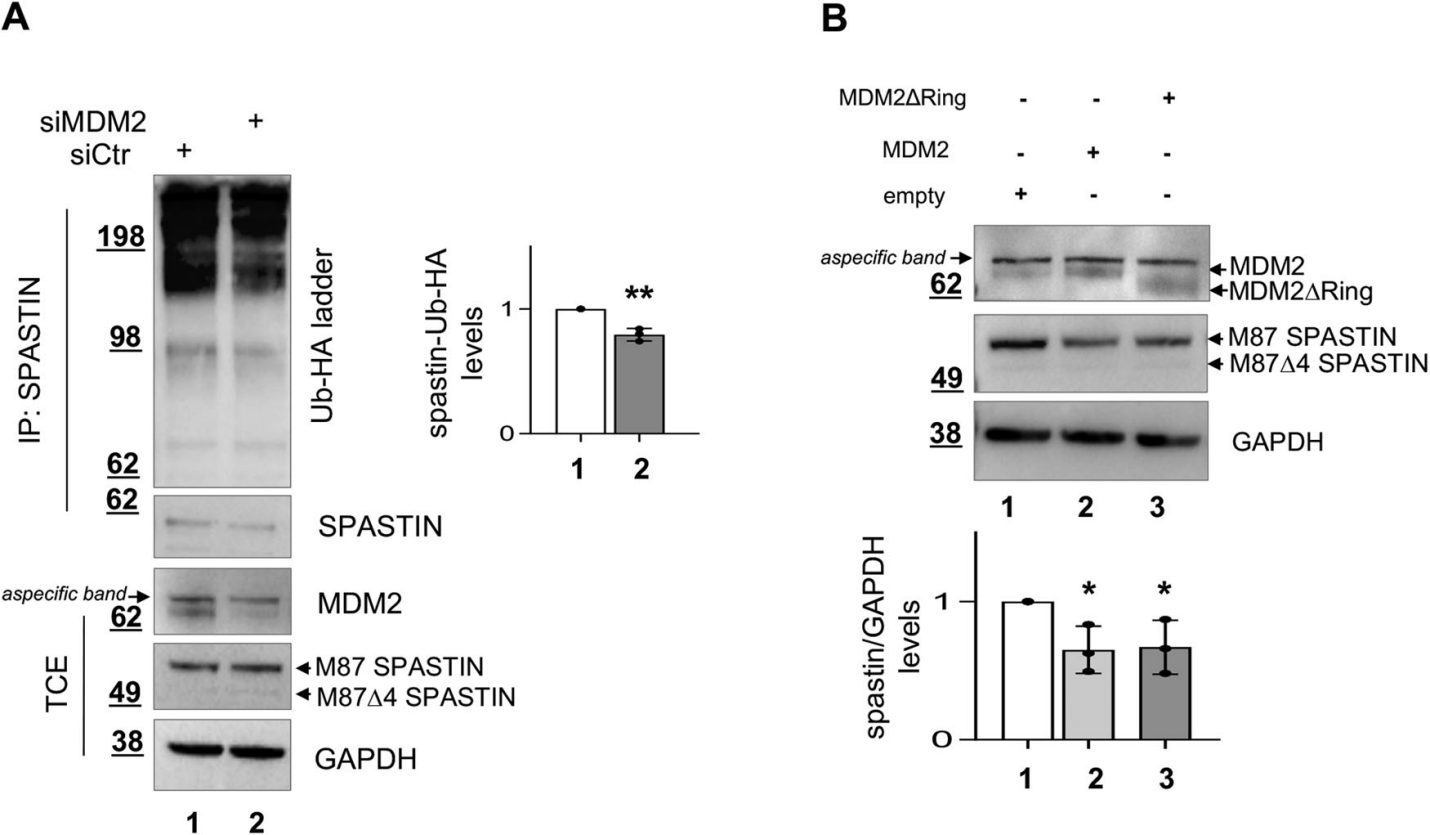
Spastin poly-ubiquitination and protein levels are regulated by MDM2 in a manner independent from its catalytic activity. **A** Spastin ubiquitination assays. HeLa cells were transfected with indicated siRNAs in combination with the vector expressing Ub-HA, and treated with MG132 for 8 h before lysis. TCEs were immunoprecipitated with anti-spastin Ab and analyzed by WB 24 h post transfection. Spastin-Ub-HA ladder intensity was normalized by the intensity of spastin in the IP fractions and reported relative to siCtr cells as mean ± SD, *n* = 3. **P < 0.01, unpaired *t*-test. **B** H1299 cells were transfected with either an empty vector or a MDM2– or MDM2ΔRing-expressing vector and analyzed 24 h post transfection by WB. Representative WB is shown and spastin quantification is reported as mean ± SD, *n* = 3. *P < 0.05, unpaired *t*-test.

Finally, we performed in cellulo spastin ubiquitination assays [7, 6] and found that siMDM2 cells show reduced levels of spastin poly-ubiquitination compared to control cells (Fig. 3A) suggesting that MDM2 regulates spastin level through its poly-ubiquitination. Next, we compared the effect on spastin protein levels induced by overexpressing MDM2 full-length and MDM2 deletion mutant lacking the E3 Ubiquitin ring domain (MDM2ΔRing). Compared to control cells transfected with an empty vector, similar reductions of spastin levels were observed in cells expressing MDM2 wild-type and in those expressing the catalytically inactive MDM2ΔRing mutant (Fig. 3B) indicating that the MDM2 catalytic activity is dispensable for spastin regulation.

Taken together, these results show that MDM2 physically interacts with spastin and regulates its poly-ubiquitination in a catalytic-independent manner.

### Targeting MDM2 rescues the levels of spastin and reduces cytokinesis and receptor-sorting defects in spastin-deficient cells

**Fig. 4:**
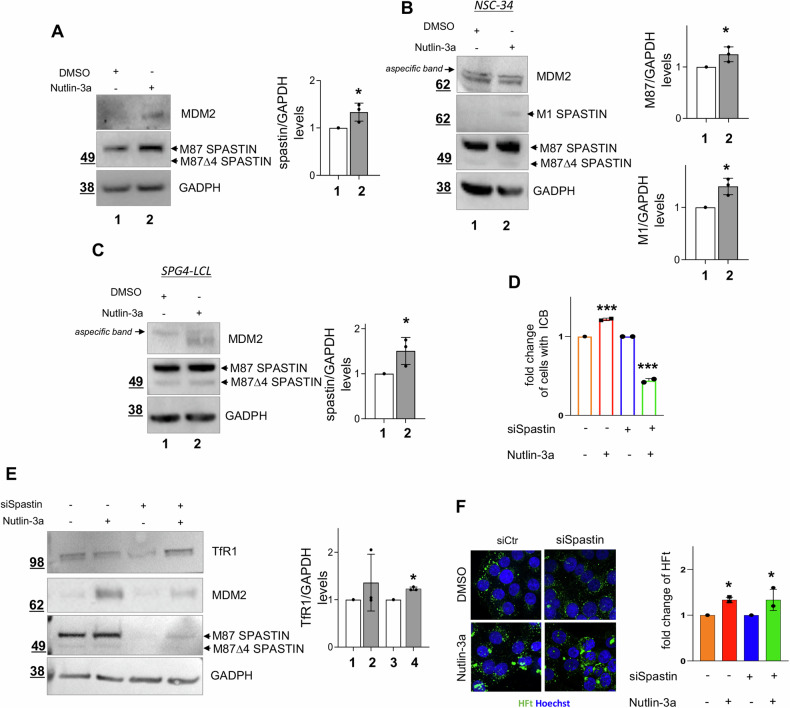
Nutlin-3a treatment increases spastin levels and reduces defects in spastin-deficient cells. **A** HeLa cells were treated with 10μM Nutlin-3a or its solvent DMSO and analyzed by WB 16 h post treatment with indicated Abs. Spastin quantification is reported as mean ± SD, *n* = 3. *P < 0.05, unpaired t-test. **B** Motor neuron-like NSC-34 cells were treated and analyzed as in (**A**). Representative WB is shown. M87 and M1 spastin quantification is reported as mean ± SD, *n* = 3. *P < 0.05, unpaired *t*-test. **C** SPG4-LCLs were treated and analyzed as in **A**. Spastin quantification is reported as mean ± SD, *n* = *3*. *P < *0.05*, unpaired *t*-test. **D**–**F** HeLa cells were transfected with 20 nM siSpastin or negative control stealth siRNAs, 48 h post siRNA transfection cells were treated with 10μM Nutlin-3a or its solvent DMSO and analyzed by WB and confocal microscopy 16 h post treatment. Cells to be analyzed by microscope were also treated with FtH-based nanocarriers for 3 h before being fixed for imaging analysis. In **D**, the percentage of cells with ICB is reported relative to solvent-treated cells, as mean ± SD. At least *n* = 1100 total cells were analyzed from 2 independent experiments. **P < 0.01, unpaired *t*-test. In (**E**), representative WB and TfR1 quantification are shown, mean ± SD, *n* = *3*. *P < 0.05, unpaired *t*-test. In **F**, representative images of fluorescent HFt uptake in the cells and its quantification are shown. Histogram, mean ± SD, *n* = 3 *P < 0.05, unpaired *t*-test. Scale bar, 10 μm.

Nutlin-3a, which is undergoing clinical trials in cancer, is a well-characterized and highly specific inhibitor of MDM2 [35, 36]. Nutlin-3a binds to MDM2 and disrupts protein-protein interactions, including that between MDM2 and p53. Upon Nutlin-3a treatment, we observed, a significant increase of spastin levels in HeLa (Fig. 4A) and in H1299 cells (Fig. S3A), in agreement with data obtained by siRNA-mediated MDM2 downregulation. In contrast, no effect on spastin levels was observed by treating with Nutlin-3a the p53 and mdm2 double knock-out (DKO) cells (Fig. S3B). The use of DKO was compulsory since there are no mdm2^−/−^ cells available, as mdm2 knock-out mice are embryonic lethal [37, 38]. However, the absence of any Nutlin-3a induced effect in the DKO cells supports the on-target effect of the MDM2-specific inhibitor in the regulation of spastin levels.

Next, we assessed the effect of Nutlin-3a on spastin in neuronal cell models commonly employed for spastin regulation and function studies [39–43], and in HSP patient-derived cells. An increase in spastin levels was observed upon Nutlin-3a treatment in human SH-SY5Y neuron-like cells (Fig. S3C) and in murine motor neuron-like NSC-34 cells (Fig. 4B). Of relevance, similar results were also observed in patient-derived SPG4-LCLs (Fig. 4C), which carries a heterozygous c751insA truncating mutation and express approximately 50% of wild-type spastin levels, with undetectable levels of truncated proteins [7]. These results indicate that pharmacological inhibition of MDM2 increases spastin levels in neuronal cells and in SPG4-HSP background, too.

Following the last observation, we investigated whether the Nutlin-3a-induced increase of spastin might reduce functional defects associated with spastin reduction. To mimic the effects of spastin haploinsufficiency, we downregulated spastin via RNAi in HeLa cells. As expected, spastin deficiency results in delayed abscission, as detected by accumulation of cells connected by ICB ([12, 14]; Fig. S3D) and TfR1 sorting defects ([15]; Fig. S3E). These defects were rescued by Nutlin-3a treatment (Fig. 4D, E). In particular, upon treatment we observed a significant decrease in cells at the cytokinesis stage (Fig. 4D) and a significant recovery in TfR1 protein levels associated with an increase of spastin levels in spastin-downregulated cells (Fig. 4E). In similar conditions, the cellular uptake of fluorescent FtH nanocarriers, whose internalization is primarily mediated by FtH/TfR1 receptor binding [17, 44], was significantly enhanced (Fig. 4F), further supporting a rescue of TfR1 sorting.

Overall, these findings indicate that the increase in spastin levels induced by Nutlin-3a may contribute to the reduction of functional defects associated with spastin reduction.

## Discussion

In this study, we have identified MDM2 as a new interactor of spastin, capable of regulating its poly-ubiquitination and protein levels. This adds a new layer of complexity to spastin regulation and expands our understanding of the factors impacting on spastin levels, which is crucial for the development of new therapeutic strategies aimed at spastin recovery in HSP patients. Our data support a HIPK2- and p53-independent mechanism of regulation of spastin levels mediated by MDM2, in a manner that is independent of its ubiquitin ligase activity. Furthermore, we show evidence supporting a direct interaction between MDM2 and spastin MIT domain, although additional domains may also be implicated.

Several possibilities arise regarding the mechanism/s underlying MDM2-mediated spastin regulation, opening a complex scenario that requires specific investigation in future explorations. For instance, MDM2 binding to spastin could promote its interaction with pro-degradative factors, such as still unknown spastin-specific deubiquitinases, or antagonize the interaction with anti-degradative factors, such as the substrate exchange factor CAND1 [7]. Additionally, MDM2 binding can affect the interaction of spastin with factors recruiting it to E3 Ubiquitin ligase complexes [45], such as DDB1-Cullin 4-Ring ubiquitin ligase complex (CRL4), that we recently shown to be involved in spastin poly-ubiquitination/degradation [6].

MDM2 represents a promising druggable target for the development of novel therapeutic strategies for the treatment of cancer and other diseases where it is implicated, beyond oncology [46, 26]. Besides p53, MDM2 interacts through its binding cleft with several proteins, including p53 family members p73 and p63, E2F-1, and hypoxia-inducible factor-1α [47–49]. Nutlin-3a binding to MDM2 impairs interactions with these proteins, displaying also p53-independent effects [47–51]. Our results indicate that Nutlin-3a leads to an increase of spastin levels in cells of diverse origins, irrespective of p53 presence. This finding requires detailed investigation but lends support to the hypothesis that this effect is likely due to Nutlin-3a-induced inhibition of MDM2-mediated protein-protein interactions other than p53. Importantly, we observed that MDM2 downregulation or Nutlin-3a-mediated inhibition can induce a recovery of spastin levels and associated functions with potential therapeutic implications for SPG4-HSP. Furthermore, targeting MDM2 for protein degradation is emerging as an additional strategy to downregulate MDM2 without the side effects of agents blocking p53-MDM2 binding, for safer and more effective treatments [26].

In terminally differentiated neuronal cells MDM2 function and regulation are largely unclear [52, 53]. A multifunctional and substrate-dependent role of MDM2 has been described in the nervous system [54, 46], but the pathways regulated by MDM2 are not yet fully understood. Here, we add MDM2 as an important regulator of spastin levels and its functions. Although this study presents limitations due to the use of cell cultures, it shows potential therapeutic implications for HSP, providing crucial a foundation for assessing the effects of MDM2 inhibition in SPG4-HSP neurons and animal models. As spastin interconnects with many other HSP-linked genes [55], continued study on MDM2 and its role in the nervous system will be crucial and could help us better understand SPG4-HSP and other HSP. Identifying the differentially regulated target genes of MDM2 in different cell populations in the nervous system could improve our understanding of these factors and open up new potential therapeutic development for HSP.

In conclusion, we found that MDM2 targeting contributes to the regulation of spastin, and may be useful to develop approaches to rescue functional defects associated with reduced spastin levels.

## Materials and methods

### Cells, culture conditions, transfection and treatments

HeLa (by N. Corbi), HeLa HIPK2 null (HIPK2-Cas9) and their parental control (Ctr-Cas9) cells (by ML Schmitz, [28]). H1299 (by D. Trisciuoglio) were cultured at 37 °C and 5% CO_2_ in DMEM GlutaMAX (Life Technologies, Waltham, MA, USA) supplemented with 10% heat-inactivated fetal bovine serum (FBS) (Life Technologies). SH-SY5Y (by G. Guarguaglini) and NSC-34 (by Dr. M Cozzolino) were cultured in DMEM-F12 1:1, supplemented with 10% FBS. The p53^−/−^mdm2^−/−^ murine embryo fibroblasts (MEF) (by G. Lozano), derived from double knockout mice, were cultured in DMEM GlutaMAX high glucose (Life Technologies) supplemented with 10% FBS. Patient-derived lymphoblastoid cells (LCL-SPG4), carrying the pathogenetic heterozygous c751insA SPG4 mutation [7], were cultured in RPMI GlutaMAX (Life Technologies) supplemented with 10% FBS.

Cells were routinely tested for mycoplasma contamination. Nutlin-3a is from Sigma-Aldrich (MERCK, Frankfurter, Germany). Fluorescent H-chain Ft (FtH) nanocarriers were obtained by incubation of HFt (1 mg ml^−1^) with 1 mM of Fluorescein-5-Maleimide (ʎex 491 nm, ʎem 518 nm; Life Technologies) in Phosphate-Buffered Saline (PBS), pH 7.0 for 2 h at room temperature (RT) under stirring in the dark. Subsequently, the sample was filtered, dialyzed, and exchanged with PBS, pH 7.4 by using 30 kDa Amicon Ultra-15 centrifugal filter devices (MERCK) to remove excess reagents. The sample was sterile filtered and stored at 4 °C in the dark. The number of dye molecules linked per protein was determined by absorbance spectroscopy, in accordance with the manufacturer’s instructions, applying the Lambert–Beer law.

### Expression vectors

The following expressing vectors were used: pCMV-MDM2, pCMV-MDM2ΔRing and pEF-Ub-HA [27], pCMV6-spastin- and spastin Δ4-DDK-MYC tagged expression vectors [14] and their entry vector pCMV6-DDK-MYC (Origene Technologies).

Vectors were transfected by using Lipofectamine LTX and Plus reagent (Life Technologies).

For GST-MDM2 and GST production, BL21 competent cells were transfected with pGEX4T-1-HsMDM2 plasmid or pGEX4T-1 as control. BL21 colonies from LB agar plate containing ampicillin, were then inoculated in LB broth with 100 μg/mL ampicillin (Sigma-Merck) and incubated at 37 °C under shaking. At the bacterial density of 0.7 (at OD600), 0.5 mM of isopropil-β-D-1-tiogalattopiranoside (IPTG) and 200 μM of ZnCl_2_ (MERCK) were added. After 2 h at 37 °C, the culture was pelleted at 3000 rpm for 20 minutes. The pellet was lysed, by sonication, with NETN buffer (50 mM Tris pH 8.3 and 150 mM NaCl) supplemented with 100 μg/mL DNase, 1 mM PMSF, 0.2 mg/mL lysozyme and 10% glycerol.

### TfR1 concentration, FtH nanocarrier uptake, and RNA interference (RNAi)

Cells were transfected with spastin or control siRNAs, and total cell TfR1 levels and FtH nanocarrier uptake were measured 72 h post transfection, as in ref. 15 For pharmacological inhibition of MDM2, cells were treated with Nutlin-3a or its solvent DMSO 56 h after transfection with spastin or control siRNAs. The cells were then harvested for analysis or treated with fluorescent FtH nanocarries [17, 44] for 3 h before being fixed for imaging analysis.

MDM2 siRNA was obtained as in ref. 27 spastin RNAi was obtained by using specific validated stealth siRNAs selected among those targeting sequences common to all spastin isoforms, as in ref. 14 SiRNAs were transfected using Lipofectamine RNAi MAX (Life Technologies) according to the manufacturer’s instructions.

### RT-PCR

RNA extraction and real-time RT-PCR were performed as in ref. 7, and relative fold-change were determined by the 2 − ΔΔCt method using GAPDH mRNA as normalizer. All reactions were performed in triplicate. Primers for GAPDH and spastin amplification are as in ref. 7. The spastin primers amplify a region common to all spastin isoforms.

### WB and IP

Total cell extracts (TCEs) were prepared in lysis buffer (50 mM Tris-HCl pH 8, 150 mM NaCl, 0.5% sodium deoxycholate, 0.1% SDS, 1% NP40, 1 mM EDTA) supplemented with protease and phosphatase inhibitors (Roche, Basel, Switzerland). Proteins were resolved using Bolt Novex Bis-Tris Gels 4–12% gradient (Life Technologies). Immunoreactivity was determined using ECL-Prime (Amersham, GE HealthCare, Chicago, IL, USA), images acquisition and densitometric analysis was performed with Image Lab software (Bio-Rad, Hercules, CA, USA). The following antibodies (Abs) were employed: anti-GST #sc-138 (1:1000), anti-GAPDH #sc-32233 (1:1000), anti-vinculin #sc-73614 (1:1000), anti-spastin sp3G11/1 #sc-53443 or sp6c6 #sc-81624 (1:200; the two Abs were indifferently used because they produce comparable results) by Santa Cruz Biotechnology (Dallas, TX, USA); anti-spastin rabbit polyclonal Ab #PA5-53581 (1:1000) by Life Technologies was used by WB to detect immunoprecipitated spastin; anti-HA #11583816001 (1:1000) mouse monoclonal Ab by Roche; anti-TfR1 H68.4 #13-6800 (1:1000) by Life Technologies, anti-MDM2 (a mix of 2A10 Ab, 1:50, kindly provided by A. Levine, and Ab1, 1:100, Oncogene Research Products, La Jolla, CA,USA); anti-p53 (DO-1, 1:1000 by Sigma-Aldrich); anti-DDK #TA50011 (1:1000) by Origene Technologies (Rockville, MD, USA); anti-HIPK2 rat monoclonal Ab (1:500; gift by ML Schmitz); anti-HRP-conjugated goat anti-mouse #7076 and anti-rabbit #7074 by Cell Signaling Technology (Danvers, MA, USA) and HRP-conjugated goat anti-rat #7077S by Santa Cruz Biotechnology. Full length uncropped original WB are reported in the ‘Supplemental Material WB’ file.

### In vitro binding assay

GST pull-down and binding assay were performed as in ref. 14 In particular, GST and GST-MDM2 were expressed as previously described. They were then incubated with 50 ng of spastin M1/M87 or M1/M87 Δ4 (Origene Technologies) or spastin recombinant fragment containing the MIT domain (79–298 aa; Proteintech Group, Rosemont, IL, USA) in 50 mM Tris-HCl, pH 7.5, 150 nM NaCl. Bound spastin proteins/fragments were analyzed by WB.

### Microscopy analysis

Cells seeded onto poly-L-lysine (Sigma-Aldrich, MERCK) coated coverslips, were fixed in 2% formaldehyde, permeabilized in PBS-T (PBS 0.25% Triton-X 100) 10’ at RT and marked with Hoechst (Sigma-Aldrich, MERCK) for nucleus labeling. Images were acquired using confocal spinning disk microscope (Crest X-Light V3; CrestOptics, Rome, Italy) equipped with the Kinetix sCMOS camera (Teledyne Photometrics; Tucson, AZ, USA), a 60x (oil immersion, N.A. 1.4) objective and CELESTA lasers (Lumencor; Beaverton, OR, USA). Acquisitions were performed along the z-axis for a total range of 3–4μm, with 0.6 μm z-step. Acquisitions, elaboration, deconvolution (3 iterations) and processing were performed using NIS- Elements AR (Nikon, Tokyo, Japan) with the JOBS module for automated acquisitions, and Adobe Photoshop CS 8. Images were quantified using the “general analysis” module of NIS-Elements H.C. 5.11 (Nikon) for automatic recognition of the whole field. The fluorescent FtH signal was measured on maximum intensity projections from acquired z-stacks, after correction for external background.

IsPLA was performed on cells grown on coverslips, fixed with 3.7% formaldehyde, using the Duolink PLA kit (DUO92007, Sigma-Aldrich, MERCK) according to manufacturer’s instructions. The amplification time was 120 min and the primary antibodies pair to detect the interaction was mouse monoclonal anti-MDM2 (1:50, SMP14, Santa Cruz biotechnologies) and rabbit polyclonal anti-FLAG (1:100, Sigma-Aldrich, MERCK). DNA was stained with DAPI. For quantification of isPLA fluorescence signals, images were acquired using a 60× objective (oil immersion; N.A. 1.4), along the z-axis every 0.4 μm for a range of 4 μm. The “general analysis” module of NIS-Elements H.C. 5.11 was used for automatic quantification of interaction dots in the Maximum Intensity Projection images.

### In silico structural analysis

The sequence of human spastin (Uniprot Code: Q9UBP0 SPAST_HUMAN) was used as input in PepThreader [34] to identify peptide motifs that can interact with MDM2. The crystal structure of MDM2 in complex with a short peptide of p53 (PDB Code: 3JZP, [31]) was used as structural template to model each helical peptide of spastin into the p53-binding cleft of MDM2. The Alpha-Fold2 [33] predicted structure of spastin was used to identify structured helical regions. Disordered regions of spastin and the intramembrane helix in HD domain (49–80) were excluded from the analysis. A normalized rainbow scale (0–1) based on the mean of PepThreader’s scoring functions was used to rank each peptide.

### Statistical analysis

Data analyses were performed using the GraphPad Prism software 8.0.2 (GraphPad Software, Inc). All experiments were repeated at least three times independently, unless otherwise indicated. For measurements of continuous variables, the unpaired two-sided Student’s t-test or one-way Anova for multiple comparisons were used. Statistical significance was set at *P* < 0.05 and was reported by asterisks according to the following scheme ***P < 0.001, **P < 0.01, and *P < 0.05.

## Supplementary information

Supplementary Figure S1

Supplementary Figure S2

Supplementary Figure S3

Supplementary figure legends

supplemental WB file

## Data Availability

The raw data supporting the conclusions of this article will be made available by the authors, without undue reservation.
