# Supplementary figures and images for: Targeting MDM2 affects spastin protein levels and functions: implications for HSP treatment
Source: Cell Death Discov. 2025 Feb 7;11:53. doi: 10.1038/s41420-025-02333-y (PMC11806007; doi:10.1038/s41420-025-02333-y)

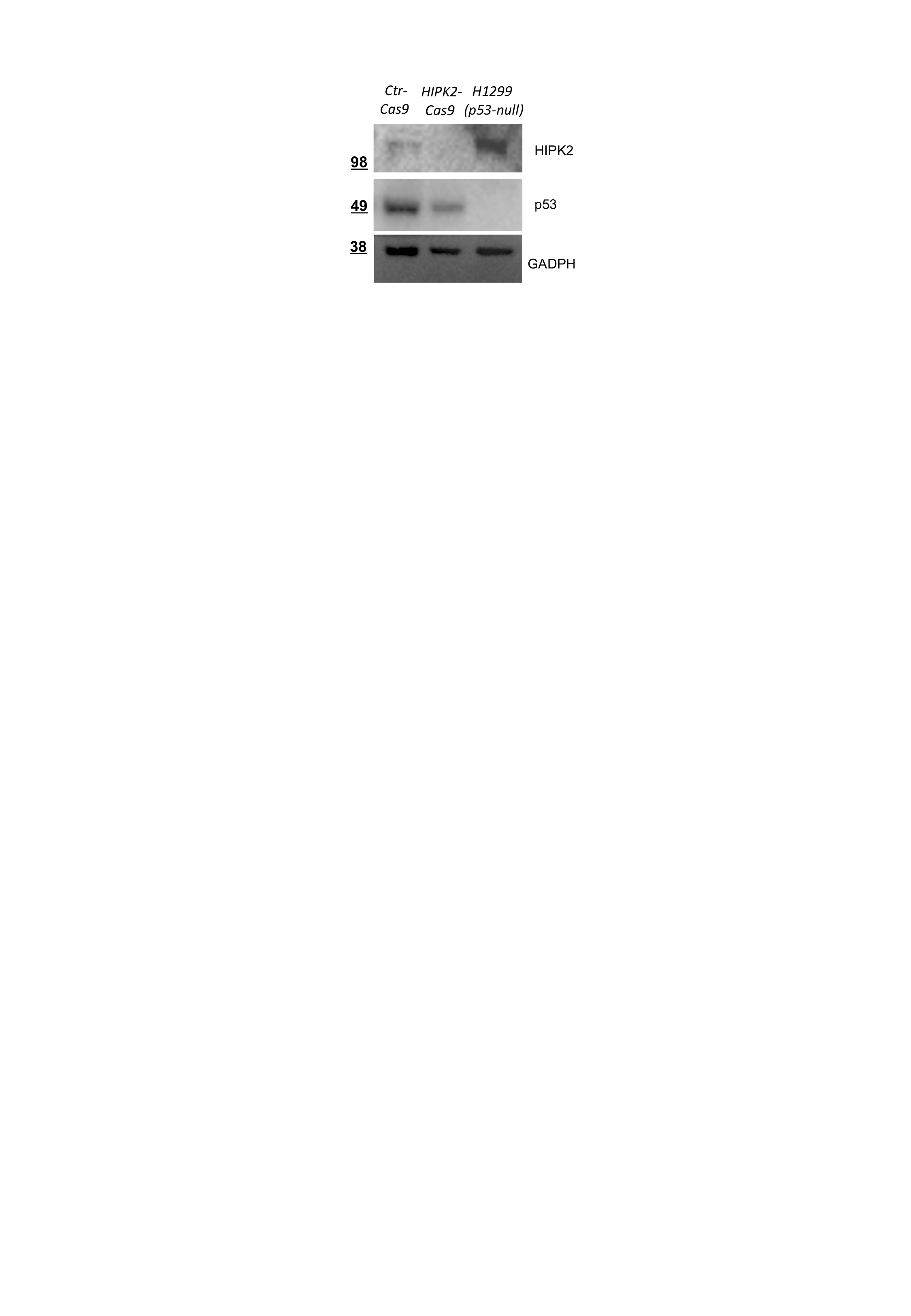

Supplement: Supplementary file 1 — Supplementary Figure S1 [file 41420_2025_2333_MOESM1_ESM.tif]

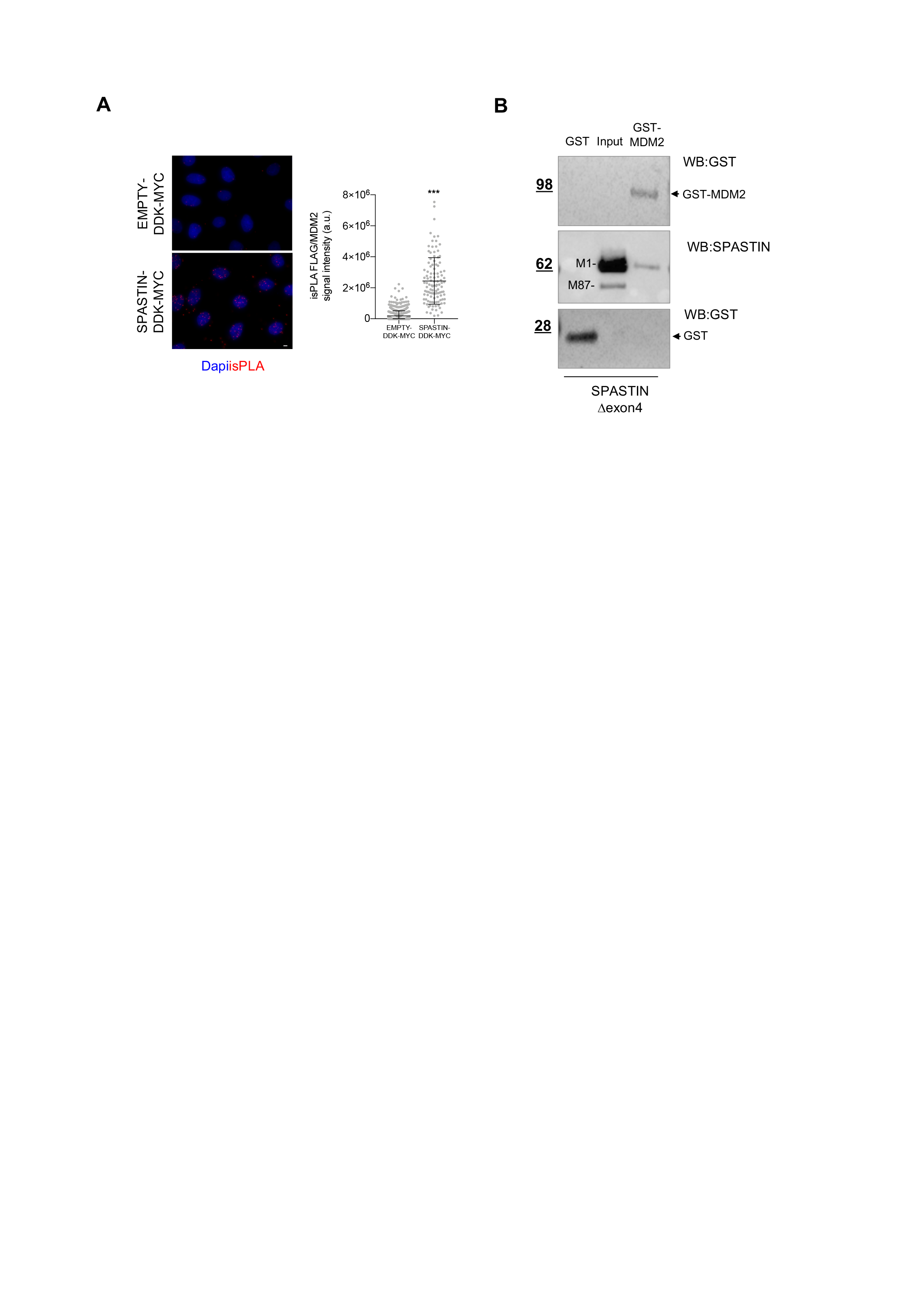

Supplement: Supplementary file 2 — Supplementary Figure S2 [file 41420_2025_2333_MOESM2_ESM.tif]

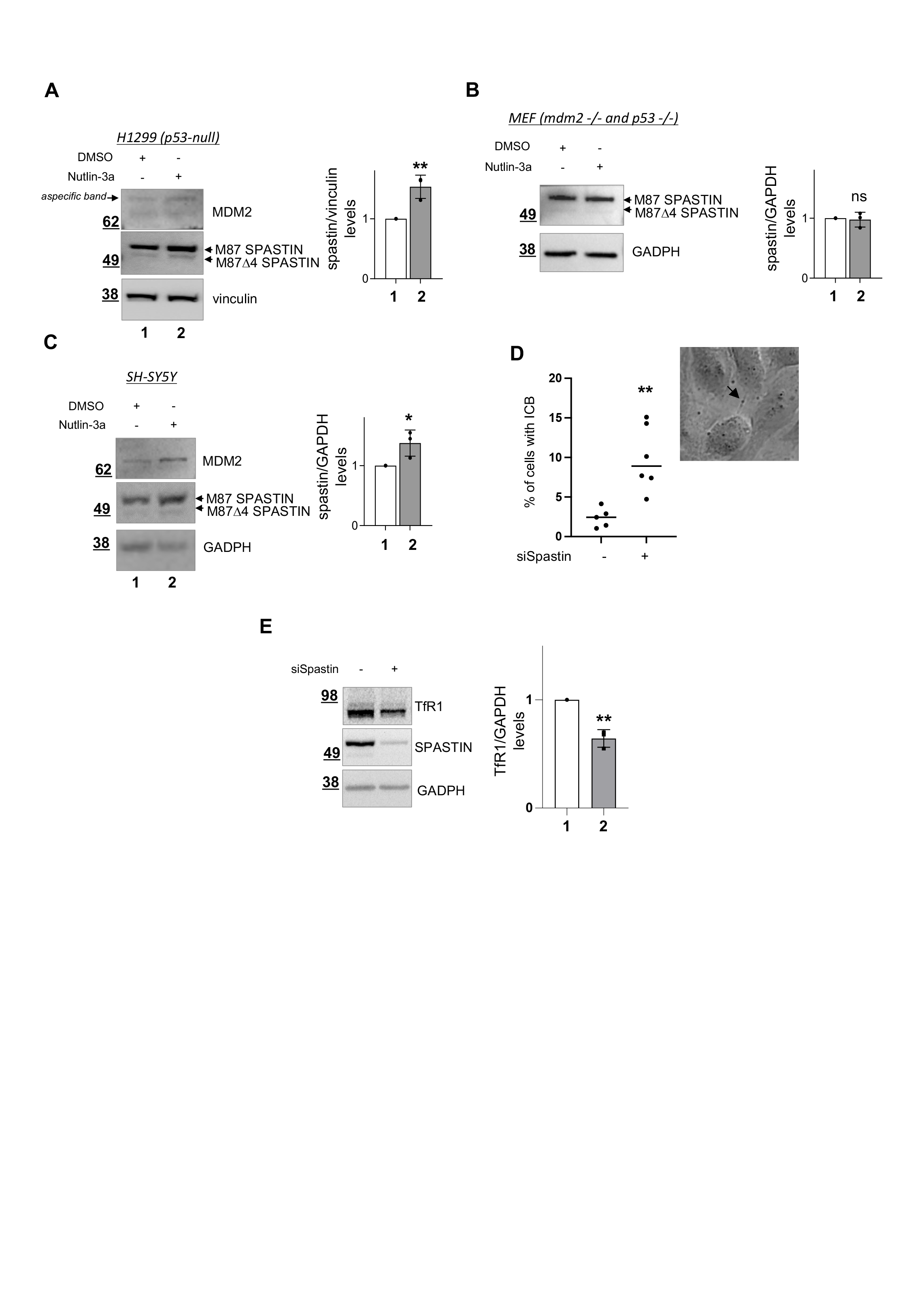

Supplement: Supplementary file 3 — Supplementary Figure S3 [file 41420_2025_2333_MOESM3_ESM.tif]

1A

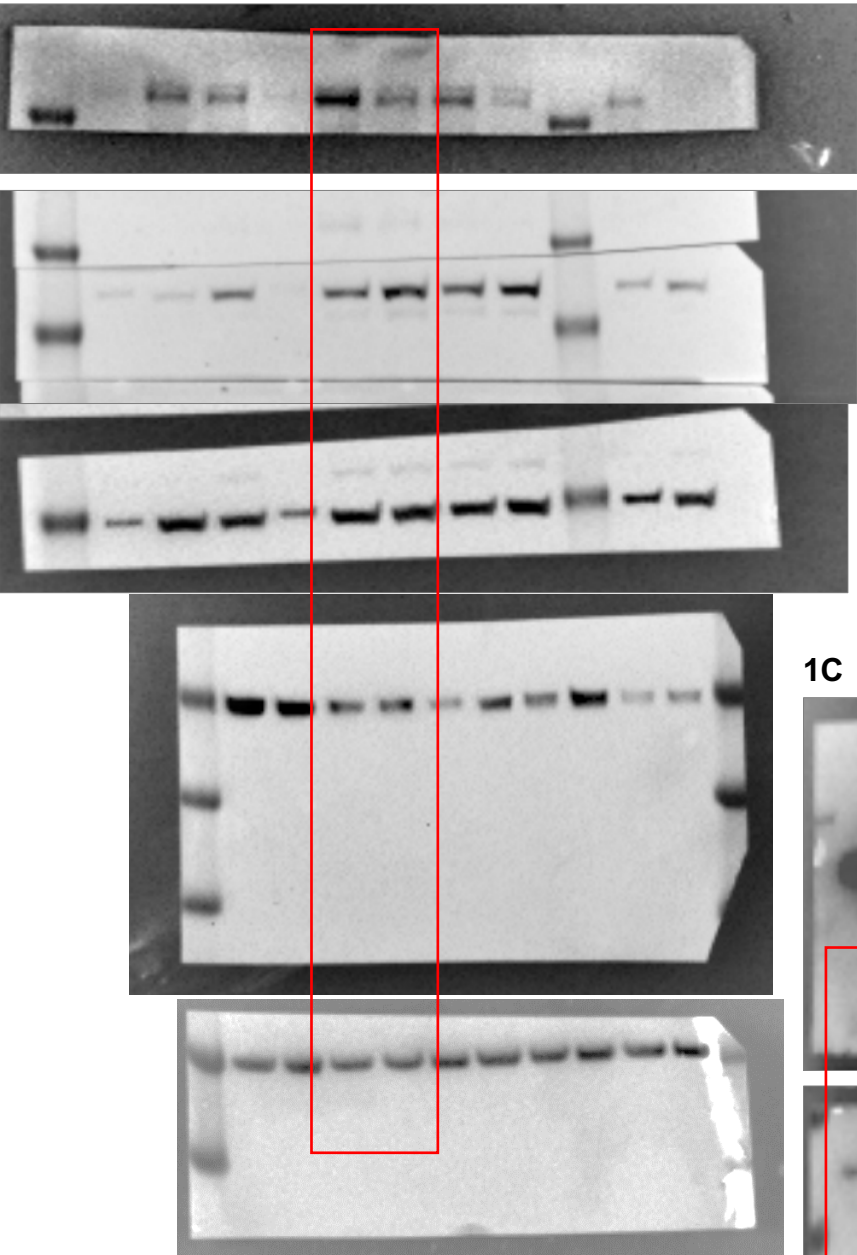

1B

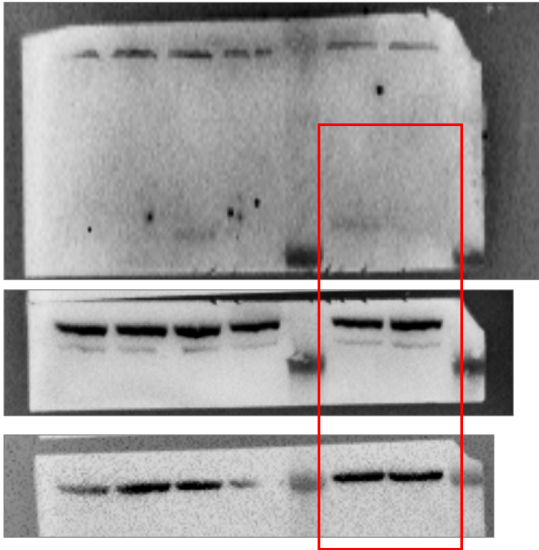

1C

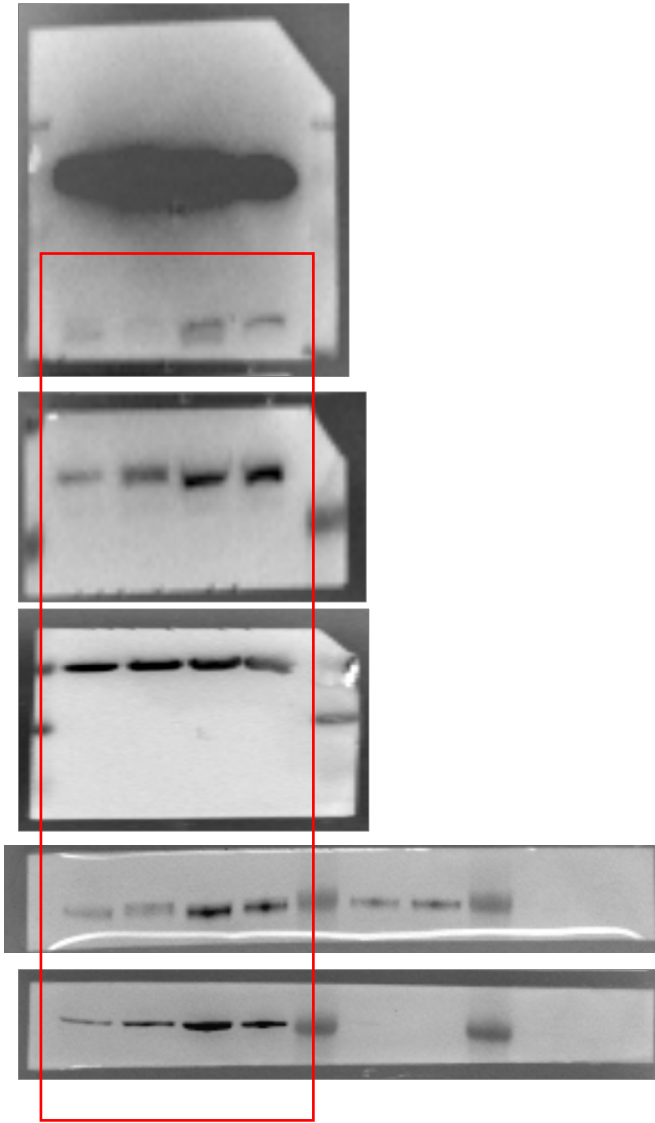

2A

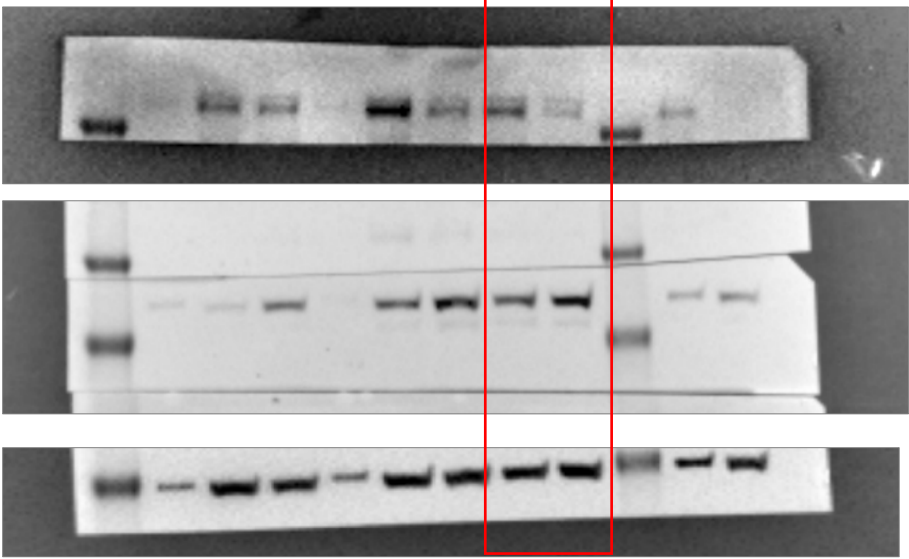

ED

2D

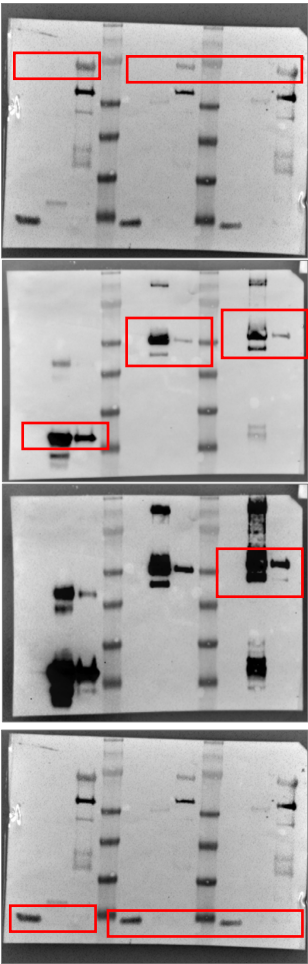

2B

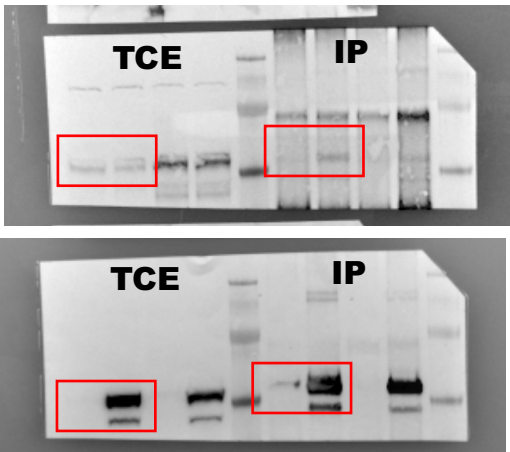

3A

TCE

IP

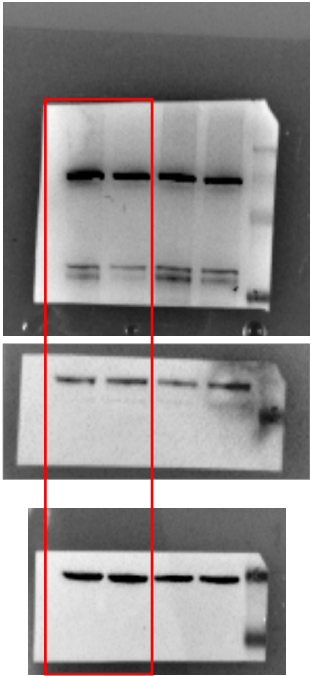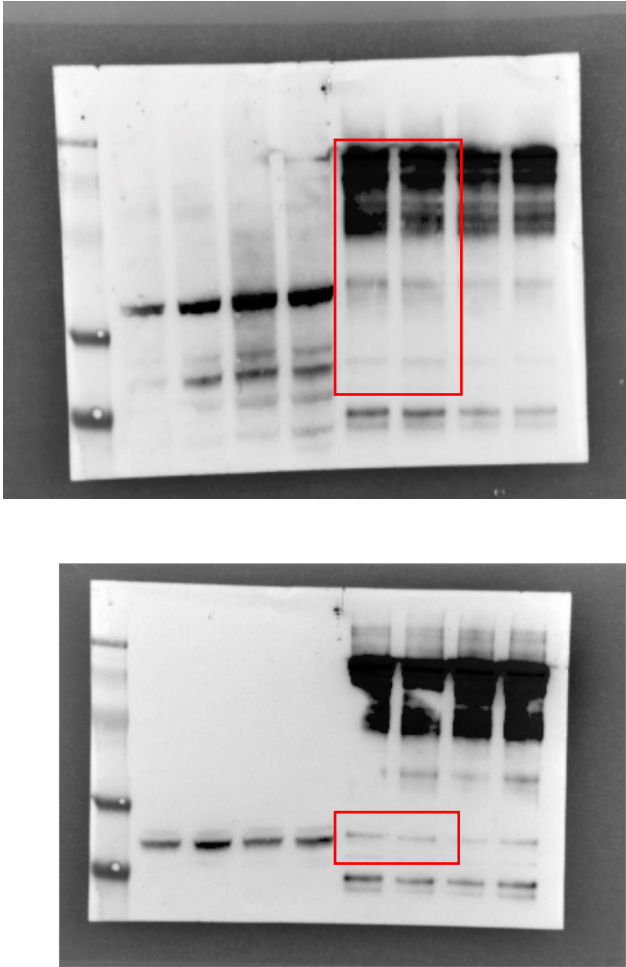

3B

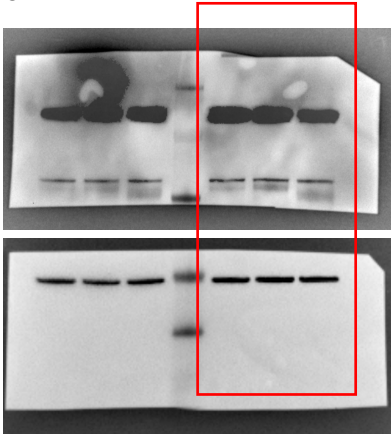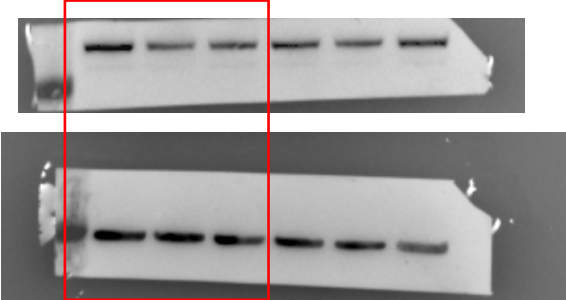

4A

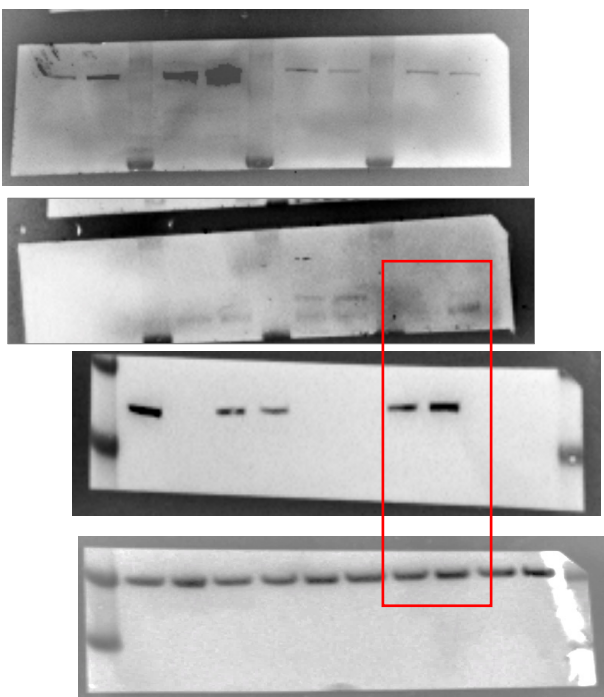

4B

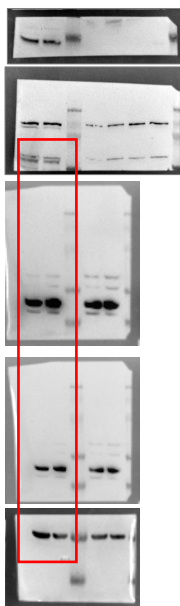

4C

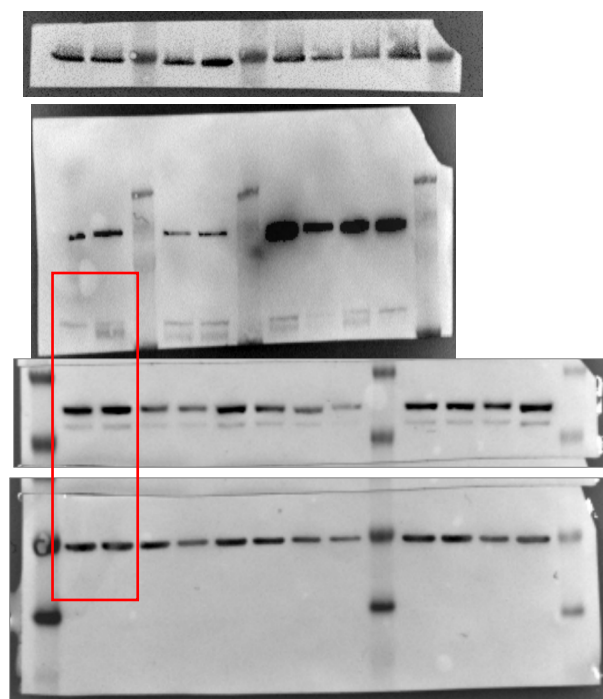

4E

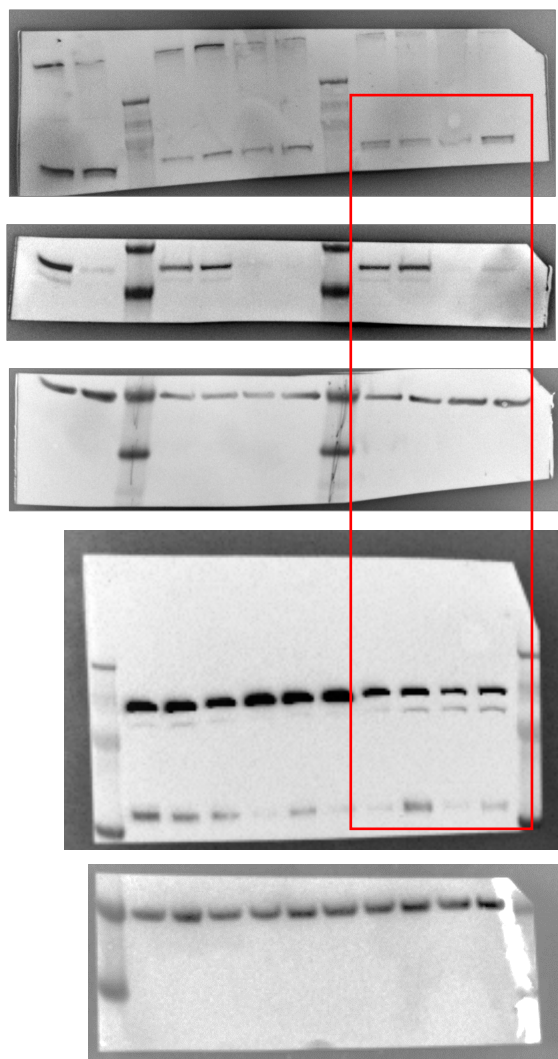

S1

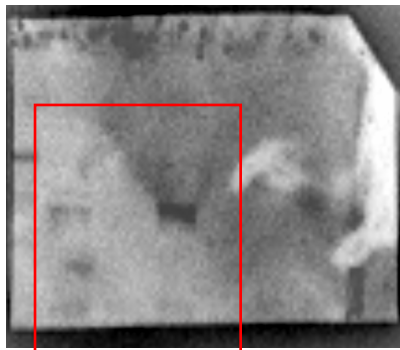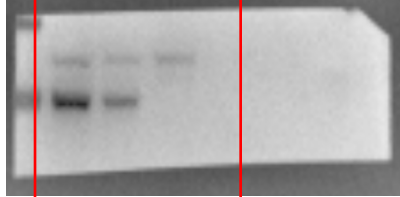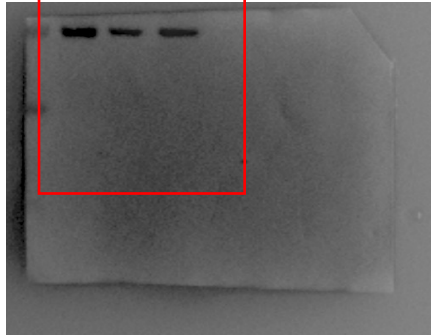

S2A

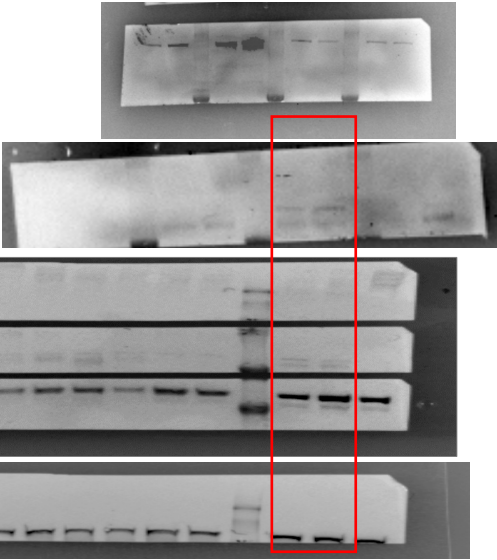

S2E

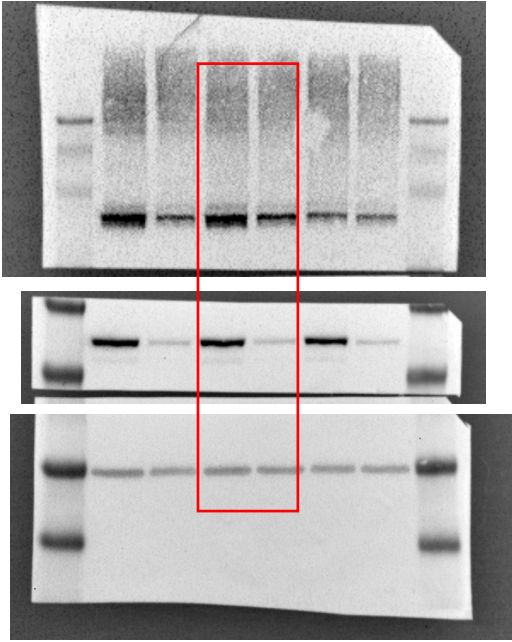

S2B

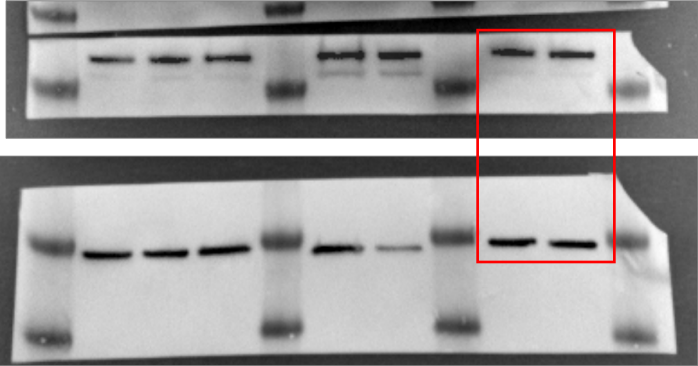

S2C

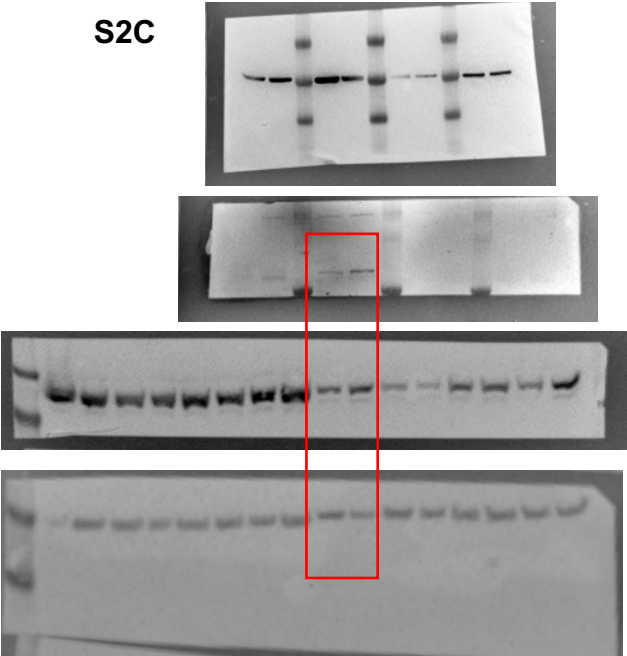

Supplement: Supplementary file 5 — supplemental WB file [file 41420_2025_2333_MOESM5_ESM.pdf]
