# Supplementary material for: Targeting MDM2 affects spastin protein levels and functions: implications for HSP treatment
Source: Cell Death Discov. 2025 Feb 7;11:53. doi: 10.1038/s41420-025-02333-y (PMC11806007; doi:10.1038/s41420-025-02333-y)
Supplement: Supplementary file 4 — Supplementary figure legends [file 41420_2025_2333_MOESM4_ESM.docx]

**Supplementary figure legends**

**Supplementary fig. S1: WB analysis of HeLa Ctr-Cas9, HeLa HIPK2-Cas9 and H1299 cells**

A representative WB with indicated Abs is shown.

**Supplementary fig. S2: MDM2/spastin interaction by isPLA and in vitro binding between MDM2 and spastin Δexon4**

**A** HeLa cell were transfected with pCMV6-DDK-MYC empty vector or pCMV6-spastin-DDK-MYC and analysed by isPLA using anti-Flag and anti-MDM2 Abs 24h post transfection. Representative images of FLAG/MDM2 interaction are shown in the indicated conditions. Quantified interaction (sum intensity of the isPLA signal per cell) is shown in the dot plot on the right. Error bars: SD; *** p<0.001, unpaired t-test. Scale bar,10 μm. **B**. GST and GST-MDM2 proteins were bacterially expressed, purified by GST pull-down, and incubated with an equal amount of indicated recombinant spastin as in the Figure 3D. Spastin binding was detected by WB. Representative WB is shown; input = 50 ng of recombinant spastin proteins M1/M87 Δexon4 (translated from the spliced isoform lacking the exon 4 that expresses spastin 1-616 Δ197-228).

**Supplementary fig. S3: Nutlin-3a treatment effect on spastin levels and analysis of functional defects in spastin-deficient HeLa cells**

**A** H1299 cells were treated and analyzed as in 4A. Representative WB and spastin quantification are shown. Spastin quantification is reported as mean ± SD, n=3, **P<0.01, unpaired t-test. **B** MEF (mdm2 -/- and p53 -/-) were treated and analyzed by WB with indicated Abs as in 4A. Spastin quantification is reported as mean ± SD, n=3, not significative (ns), unpaired t-test. **C** Neuron-like cells, SH-SY5Y were treated and analyzed as in 4A. Representative WB and spastin quantification are shown. *P<0.05, unpaired t-test.

**D,E** HeLa cells were transfected with 20 nM siSpastin or negative control stealth siRNAs and analyzed by microscopy and WB 72h post transfection. In **D**, cells were analyzed as in 4D and the percentage of cells with ICB reported as mean ± SD. **P<0.01, unpaired t-test. Representative image of cells with ICB is shown, scale bar, 10 μM. In **E**, representative WB and TfR1 quantification are shown, mean ± SD, n=3. **P<0.01, unpaired t-test
